# Ku70 affects the frequency of chromosome translocation in human lymphocytes after radiation and T-cell acute lymphoblastic leukemia

**DOI:** 10.1186/s13014-022-02113-3

**Published:** 2022-08-19

**Authors:** Zhenbo Cheng, Yupeng Wang, Lihuang Guo, Jiancheng Li, Wei Zhang, Conghui Zhang, Yangxu Liu, Yue Huang, Keqian Xu

**Affiliations:** 1 Department of Laboratory Medicine, The Third Xiangya Hospital, Central South University, Changsha, 410013 People’s Republic of China; 2 Department of Laboratory Medicine, Xiangya School of Medicine, Central South University, Changsha, 410013 People’s Republic of China; 3 Department of Medical Laboratory, Hunan Provincial People’s Hospital, The First Affiliated Hospital of Hunan Normal University, Changsha, 410005 People’s Republic of China

**Keywords:** Ku70, Chromosome translocation, Human lymphocytes, Radiation, T-cell acute lymphoblastic leukemia, Radiation damage biomarker, Therapy target

## Abstract

**Background:**

As one of the most common chromosomal causes, chromosome translocation leads to T-cell acute lymphoblastic leukemia (T-ALL). Ku70 is one of the key factors of error-prone DNA repair and it may end in translocation. So far, the direct correlation between Ku70 and translocation has not been assessed. This study aimed to investigate the association between Ku70 and translocation in human lymphocytes after radiation and T-ALL.

**Methods:**

Peripheral blood lymphocytes (PBLs) from volunteers and human lymphocyte cell line AHH-1 were irradiated with X-rays to form the chromosome translocations. Phytohemagglutinin (PHA) was used to stimulate lymphocytes. The frequency of translocation was detected by fluorescence in situ hybridization (FISH). Meanwhile, the expression of Ku70 was detected by reverse transcription-quantitative polymerase chain reaction (RT-qPCR) and western blot. Furthermore, Ku70 interference, overexpression and chemical inhibition were used in AHH-1 cell lines to confirm the correlation. Finally, the expression of Ku70 in T-ALL samples with or without translocation was detected.

**Results:**

The expression of Ku70 and frequencies of translocation were both significantly increased in PBLs after being irradiated by X-rays, and a positive correlation between the expression (both mRNA and protein level) of Ku70 and the frequency of translocation was detected (r = 0.4877, *P* = 0.004; r = 0.3038, *P* = 0.0358 respectively). Moreover, Ku70 interference decreased the frequency of translocations, while the frequency of translocations was not significantly affected after Ku70 overexpression. The expression of Ku70 and frequencies of translocation were both significantly increased in cells after irradiation, combined with chemical inhibition (*P* < 0.01). The protein level and mRNA level of Ku70 in T-ALL with translocation were obviously higher than T-ALL with normal karyotype (*P* = 0.009, *P* = 0.049 respectively).

**Conclusions:**

Ku70 is closely associated with the frequency of chromosome translocation in human lymphocytes after radiation and T-ALL. Ku70 might be a radiation damage biomarker and a potential tumor therapy target.

**Supplementary Information:**

The online version contains supplementary material available at 10.1186/s13014-022-02113-3.

## Background

Chromosome translocations are estimated to drive about 20% of cancer cases, especially hematopoietic malignancies such as T-cell acute lymphoblastic leukemia (T-ALL) [1]. It has also been reported that translocation plays a crucial role in both the onset and progression of tumorigenesis [2, 3]. The molecular mechanisms still remain unclear.

DNA double-strand breaks (DSBs) are the initial events of chromosome translocation [4, 5]. Inappropriate end-joining events caused by error-prone DSBs repair can cause the formation of translocation, thus resulting in genomic instability and cell death [6], even oncogenic transformation [7].

Ku70, encoded by DNA repair gene *XRCC6,* is one of the key factors of DSBs repair. During the DSBs repairing, the Ku heterodimer, namely Ku70 and Ku80, binds to the broken DNA as the first component of non-homologous end joining (NHEJ) [8]. Alternative end joining (Alt-EJ) is another important pathway to repair DSBs with high error propensity [9], and the PARP1 is a key factor to Alt-EJ [10]. A previous study has demonstrated that Ku70 facilitates NHEJ pathway and inhibits alt-EJ pathway [11]. In addition, it was also proposed that NHEJ factors such as Ku70 suppress the mobility of the broken ends and thus inhibit chromosome translocation [12, 13]. Mouse embryonic fibroblasts deficient in Ku70 also showed high frequency in translocations [14].

However, whether Ku70 is directly related to the frequency of translocation in human lymphocyte cells is largely unknown. In the present study, the potential association of Ku70 and translocation was investigated by combining the human primary lymphocyte cells and lymphocyte cell line after radiation for the first time, and finally verified in T-ALL samples. This study aimed to reveal the underlying association of Ku70 and translocation in human lymphocyte cells after radiation and provide the basis for the mechanism of translocation and tumor radiotherapy.

## Materials and methods

### Subjects

The study involved 38 clinic samples (6 initial T-ALL with translocation, 19 initial T-ALL with normal karyotype and 13 non-tumor samples were obtained from remission bone marrow aspirates with less than 1% leukemic cells as normal controls) from Xiangya Hospital of Central South University (Changsha, China) from June 2018 to May 2021. At the same time, another 12 age-matched healthy volunteers who had physical examinations at the same hospital were recruited. All the volunteers had no X-ray radiation, chemical poisons touch, smoking history and karyotype abnormalities. All participants signed the informed consent form before participating in the study, and the study was approved by the Ethics Committee of the Third Xiangya Hospital of Central South University. The accession number for this approval was Quick19159. The details of all patients are presented in Additional file 1: Table S1. Overall, the gender, age, ethnicity, and occupations were similar between T-ALL and remission patients. No statistically significant differences were identified (data not shown).

### Cells culture and treatments

Peripheral blood was collected from each healthy volunteer and treated with X-rays. And 0.5 mL whole blood from each group was added into the lymphocytes culture medium (Le Chen, Shanghai, China) at a humidified atmosphere of 37 ℃ and 5% of CO2 in air. 50 μg/mL phytohemagglutinin (PHA) was used to stimulate lymphocytes into exponentially growing [15]. Furthermore, the peripheral blood lymphocytes (PBLs) were separated from the rest whole blood and suspended in the lymphocytes culture medium for further culturing. The lymphocyte cell lines AHH-1 were bought from ATCC (American Type Culture Collection, CRL-8146, USA, STR profiling: Amelogenin: X,Y; CSF1PO: 10; D13S317: 11,13; D16S539: 10; D5S818: 12,13; D7S820: 10,11; THO1: 6,9.3; TPOX: 8,9; vWA: 18,19). The cell lines were cultured in RPMI 1640 (Thermo Fisher Scientific, USA) supplemented with 10% FBS (Thermo Fisher Scientific, USA). The cultures were incubated at 37˚C in a humidified atmosphere containing 5% of CO2.

### Bone marrow Karyotype

A standard 72 h lymphocyte culture of bone marrow (1–2 mL) from each patient was performed to produce Metaphases for karyotyping. G banding was performed by a trypsin pretreatment of chromosomes followed by Giemsa staining. Chromosomes’ analysis was done using MetaSystems Ikaros (ZEISS, Germany) and karyotypes were reported based on International System for Human Cytogenetic Nomenclature [16]. Karyotype analysis was conducted using at least 20 Metaphases for each sample. The number was expanded to 100 metaphases in case of suspected mosaicism.

### RNA extraction, cDNA preparation and reverse transcription-quantitative polymerase chain reaction (RT-qPCR)

According to the manufacturer's instructions, cellular RNAs were extracted using TRIZOL reagent (Takara Bio, Japan). The RNA quality was assessed using a Nanodrop One (Thermo Fisher Scientific, USA) and agarose gel electrophoresis. cDNA was generated from 2 µg of total RNA using M‑MLV reverse transcriptase (Invitrogen, USA) with random primers. Quantitative PCR (qPCR) was performed on triplicate samples in a reaction mix of SYBR‑Green (Takara Bio, Japan) using the ABI 7500 Real‑Time PCR System (Applied Biosystems, USA). The conditions of PCR denaturation, annealing and extension were 94˚C 30 s, 37˚C 30 s, and 72˚C 45 s, respectively. Relative expression of Ku70 was normalized to GAPDH using the 2^−ΔΔCq^ method [17]. The primers were synthesized by Sangon Biotech Co. Ltd. (Shanghai, China). Each RT‑qPCR reaction was performed in triplicate. The primer sequences were as follows: Ku70 forward, 5′-GGTTTCAAGCCGTTGGTACTGC-3′ and reverse, 5′-CTCCAGACACTTGATGAGCAGAG-3′; GAPDH forward, 5′-ACCACAGTCCATGCCATCAC-3′ and reverse, 5′-TCCACCACCCTGTTGCTGTA-3′.

### Western blot analysis

Total protein from cells was acquired using lysis buffer (KeyGen Biotech, Nanjing, China). Pierce BCA Protein Assay Kit (Thermo Fisher Scientific, USA) was used to calculate the protein concentration, and 30 μg of protein was loaded on an 8% of SDS-PAGE gel at 80 V for 1.5 h and transferred to 0.45 μm PVDF membranes (Millipore, USA). The membranes that were blocked with TBS‑0.05% Tween 20 (TBST) containing 5% skimmed milk for 2 h at room temperature were incubated with primary rabbit antibodies (Cell Signaling Technology, all diluted1:1,000) overnight at 4˚C and secondary HRP-conjugated goat anti‑rabbit antibody (Cell Signaling Technology, 1:5000 diluted) for 1 h at room temperature in sequence. The protein blots were then visualized using an ECL kit (Beyotime Institute of Biotechnology, China). Semi‑quantitative analysis of proteins was carried out with Image Lab (Bio-Rad, USA). GAPDH was used as the loading control, and each western blot analysis experiment was repeated three times.

### Ku70 interference and overexpression

According to the manufacturer's instructions, transfection was performed using Lipofectamine® 3000 transfection reagent (Thermo Fisher Scientific, USA). AHH-1 cells were transfected with shRNA of Ku70 (Genebio, Shanghai, China), targeted with sequence AACCAAGACCCGGACCTTTAA (Ku70-shRNA) or scrambled shRNA (negative control) targeted with TTCTCCGAACGTGTCACGT following the manufacturer’s instructions to reduce the intracellular Ku70 levels. pcDNA3.1 or pcDNA3.1+Ku70 (Genebio, Shanghai, China) was transfected into cells to overexpress Ku70. Transfection efficiency was assessed by western blotting after 72 h culturing. All cells were harvested and suspended in fresh lymphocytes culture medium, then irradiated by different dose X-rays, and followed by another 72 h culturing. The expression of Ku70, PARP1 and the frequency of chromosome translocations in the cells were explored by western blot and fluorescence in situ hybridization, respectively.

### Chemical inhibition

PARP1 inhibitor Olaparib (Abmole, USA) at 5 μM or 0.1% DMSO (solvent control) was added into the cells culture medium 3 h before X-rays. After 72 h culturing, the expression of Ku70, PARP1 and the frequency of chromosome translocations in cells were explored by western blot and fluorescence in situ hybridization, respectively.

### Fluorescence in situ hybridization (FISH)

FISH was used to detect the chromosome translocations in human PBLs and cell lines after X-rays as previously described [18]. Metaphases were harvested after co-cultured with colchicine for 2 h. Chromosomes 1 and 4 were painted green by in situ hybridization with composite probes labeled with SYBR green (Cytocell, UK), and chromosomes 2 were painted red by in situ hybridization with composite probes labeled with Rhodamine B (Cytocell, UK). The observed frequency of translocations (*F*_p_) detected by FISH represents the frequency between painted chromosomes 1, 2 and 4 and the remaining counterstained chromosomes. To compare *F*_p_ with the values for translocations detected by the conventional method that detects the aberrations involving the entire chromosome set, it is necessary to estimate the genome-equivalent frequency of translocations (*F*_G_). Thus, since the fraction of the total genomic DNA content represented by painted chromosomes 1, 2 and 4 to the total genome is 0.228 for males and 0.224 for females. *F*_p_ was multiplied by 2.771 for males and 2.806 for females to estimate *F*_G_. The basic method used is essentially described by Pearce et al. [19]. A total of 400 metaphase splitting images were observed for each sample by three observers. The experiments were repeated three times.

### Comet assay (neutral condition)

The relative amount of DNA double-strand breaks (DSBs) was detected by neutral comet assay in PBLs, as previously described [20]. Slides prepared were evaluated using a fluorescence microscope and the CASP software (CASP, Wroclaw, Poland). The data were expressed as tail intensity (% Tail DNA), and the experiments were repeated three times.

### Statistical analysis

An analysis was performed by GraphPad Prism 7.0 (GraphPad Software, San Diego, CA). Measurement data were expressed as the mean ± standard deviation (SD). Two-group comparisons were analyzed by a two-tailed Student's t-test. One-way ANOVA and two-way ANOVA were used for multiple comparisons. *P* < 0.05 was considered to be statistically significant.

## Results

### There was a dose- and time-response of radiation on Ku70 in human PBLs

**Fig. 1:**
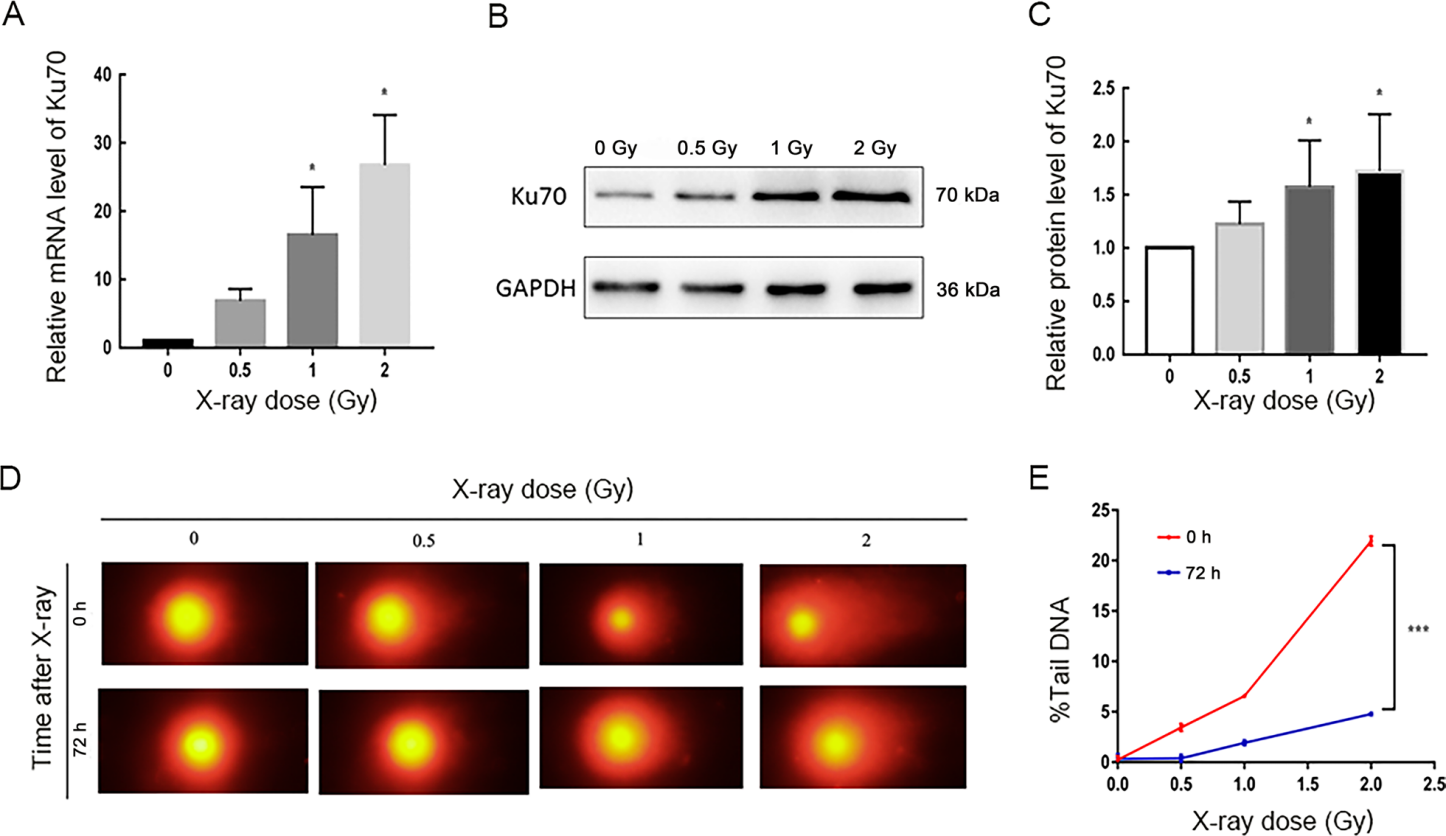
Dose- and time-response of radiation on DSBs and Ku70 in human PBLs. **A** RT-qPCR analysis of Ku70 mRNA. The expression of Ku70 mRNA accumulated in 1 Gy and 2 Gy X-irradiated PBLs. **B, C** Representative images and western blot analysis. Ku70 also showed the increment in protein level after 1 Gy and 2 Gy X-ray radiations. **D** Neutral comet assay. PBLs were isolated and subjected to 0.5 Gy, 1 Gy and 2 Gy X-ray radiations and then subjected to the neutral comet assay after 0 h and 72 h, respectively. **E** Results of Comet images analyzed. All data are presented as mean value ± SD of three independent experiments. *indicates that *P* < 0.05, and ***denotes that *P* < 0.001 compared with the control

The expression of Ku70 in human peripheral blood lymphocytes (PBLs) after X-rays was explored first. Figure 1a shows that the expression of Ku70 mRNA in PBLs accumulated in 1 Gy and 2 Gy X-rays (*P* < 0.05). Besides, Ku70 also displayed the increment in protein level after 1 Gy and 2 Gy X-rays (Fig. 1b, c, *P* < 0.05). Radiation exposure in 0.5 Gy did not change the expression of Ku70 in human PBLs (Fig. 1a–c, *P* > 0.05). Neutral comet assay revealed that after exposure to 0.5 Gy, 1 Gy and 2 Gy radiation, the DNA damage extent markedly increased in PBLs immediately (0 h), and only 1 Gy and 2 Gy groups still a markedly increased at 72 h (Table 1, *P* < 0.01, *P* < 0.001 respectively). The PBLs DNA damage at 72 h reduced obviously in all groups compared with that at 0 h (Fig. 1d, e, *P* < 0.001).

**Table 1:** Human peripheral blood lymphocytes X-ray dose response (n = 3)

Time after treated with X-ray	0 h				72 h			
X-ray dose (Gy)	0	0.5	1	2	0	0.5	1	2
% Tail DNA^a^	0.01	3.64*	6.62***	22.18***	0.03	0.23	2.07**	4.85***
SD^b^ (%)	0.54	7.80	1.00	11.87	1.62	1.30	5.03	2.33

^a^% Tail DNA: Mean percentage of total DNA, calculated from averages of three repeats

^b^SD: standard deviation. One-way ANOVA was performed to compare each treated dose to negative controls (*P < 0.05, **P < 0.01, ***P < 0.001)

### The frequency of translocations showed a positive correlation with levels of Ku70

**Fig. 2:**
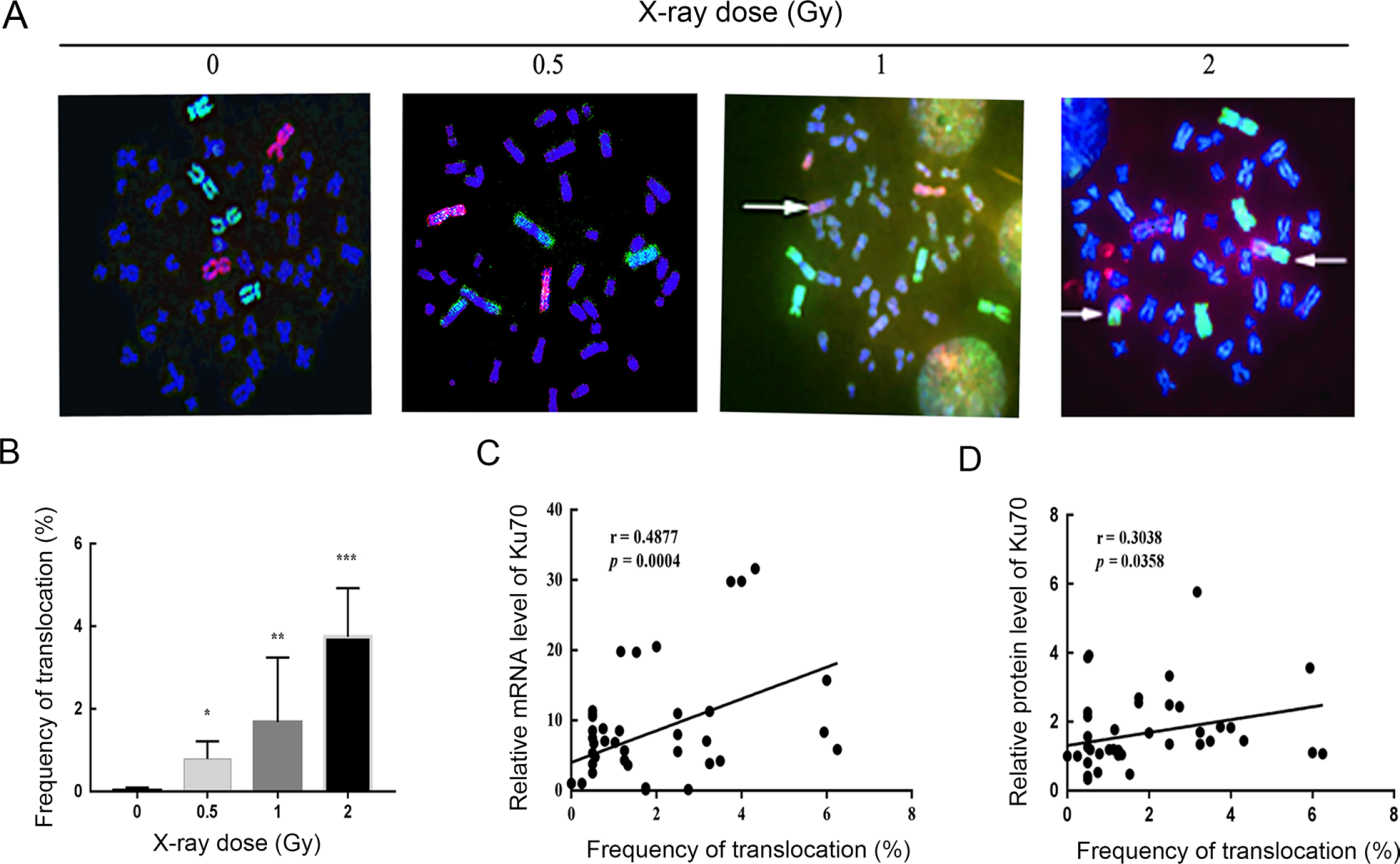
Correlation between the frequency of chromosome translocations and the level of Ku70. **A** Representative images of photomicrographs showed FISH painted human chromosome 1, 4 (green) and 2 (red) in metaphase lymphocytes after X-rays. Chromosomes translocations were displayed with the arrows. **B** FISH analysis showed that X-ray radiations increased the frequency of chromosome translocations of PBLs. **C** Pearson correlation analysis of chromosome translocations and Ku70 mRNA showed a significant positive correlation of the frequency of chromosome translocations versus the expression of Ku70 mRNA (Pearson r = 0.4877; *P* = 0.004). **D** Pearson correlation analysis of chromosome translocations and Ku70 protein level displayed a significant positive correlation of the frequency of chromosome translocations versus the expression of Ku70 protein (Pearson r = 0.3038; *P* = 0.0358). *indicates that *P* < 0.05, and **represents that *P* < 0.01. ***refers to that *P* < 0.001

To reveal whether Ku70 is related to the frequency of translocation in human primary lymphocyte cells, PBLs were irradiated with an increasing dose of X-rays and left to repair for 72 h. Chromosome translocations were observed by FISH (Fig. 2a). The whole-genome translocation frequencies in different groups were illustrated in Fig. 2b. The frequency of translocations detected after 0.5 Gy, 1 Gy and 2 Gy X-rays was significantly higher than that in non-irradiated control (*P* < 0.05, *P* < 0.01 and *P* < 0.001 respectively). Furthermore, Pearson correlation analysis was performed to explore the relevance of Ku70 and chromosome translocations in human PBLs after X-rays. The frequency of chromosome translocations proved a moderate positive correlation with the level of Ku70 mRNA (Fig. 2c, r = 0.4877, *P* = 0.0004) and a weak positive correlation with the level of Ku70 protein (Fig. 2d, r = 0.3038, *P* = 0.0358).

### The cell cycle lymphocytes irradiated in has little influence on translocation

**Fig. 3:**
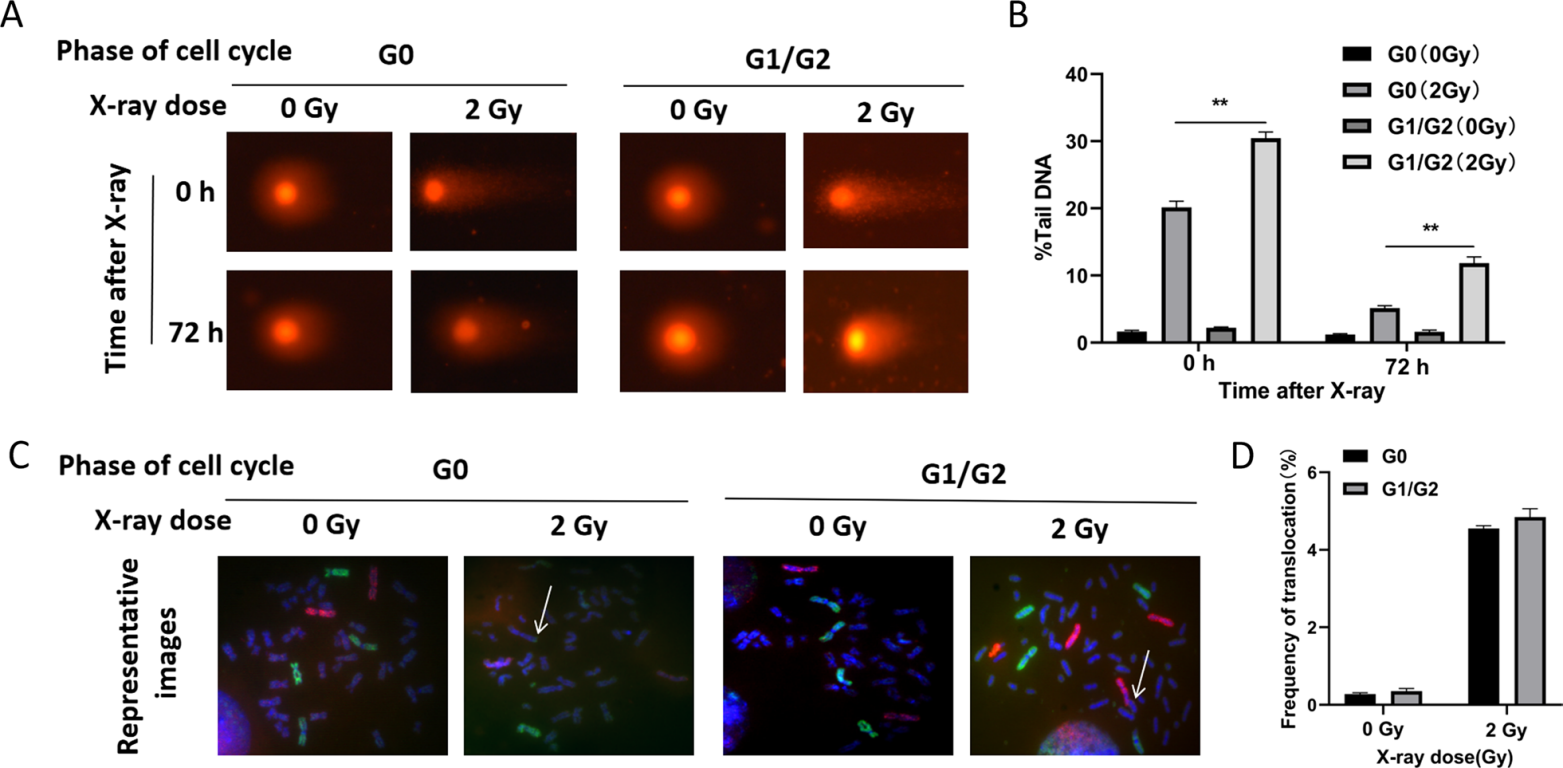
Influence of the cell cycle on DSBs and translocations in PBLs after irradiated. **A** Neutral comet assay. PBLs in G0 or G1/G2 were subjected to 0 Gy or 2 Gy X-ray radiations and then subjected to the neutral comet assay after 0 h and 72 h, respectively. **B** Results of Comet images analyzed. All data are presented as mean value ± SD of three independent experiments. **denotes that *P* < 0.01. **C** Representative images of photomicrographs showed FISH painted human chromosome 1, 4 (green) and 2 (red) in metaphase lymphocytes after X-rays. Chromosomes translocations were displayed with the arrows. **D** Results of FISH analyzed. There was no statistical difference in translocation frequency between cells in G0 phase and cells in G1/G2 phase

Previous reports used exponentially growing cells to measure translocations most in G1 or G2 phase irradiated cells [21–23]. To approximate the real clinical radiotherapy situation, lymphocytes were irradiated in the G0 phase of the cell cycle in our study. However, whether different cell cycle phases affects the translocation outcome remains unclear. Our additional experiments results showed that G1/G2 phase cells is more sensitive to irradiation compared with cells in G0 phase (Fig. 3a, b, *P* < 0.01), while little influence was found on the translocation outcome across different cell cycles (Fig. 3c, d, *P* > 0.05). Although it has been reported that G2 phase is more prone to translocation [24], we still believe there was little observed discrepancy on translocation outcome in our study for the differences in experimental treatment and cell type.

### Ku70 interference decreased the frequency of chromosome translocations

**Fig. 4:**
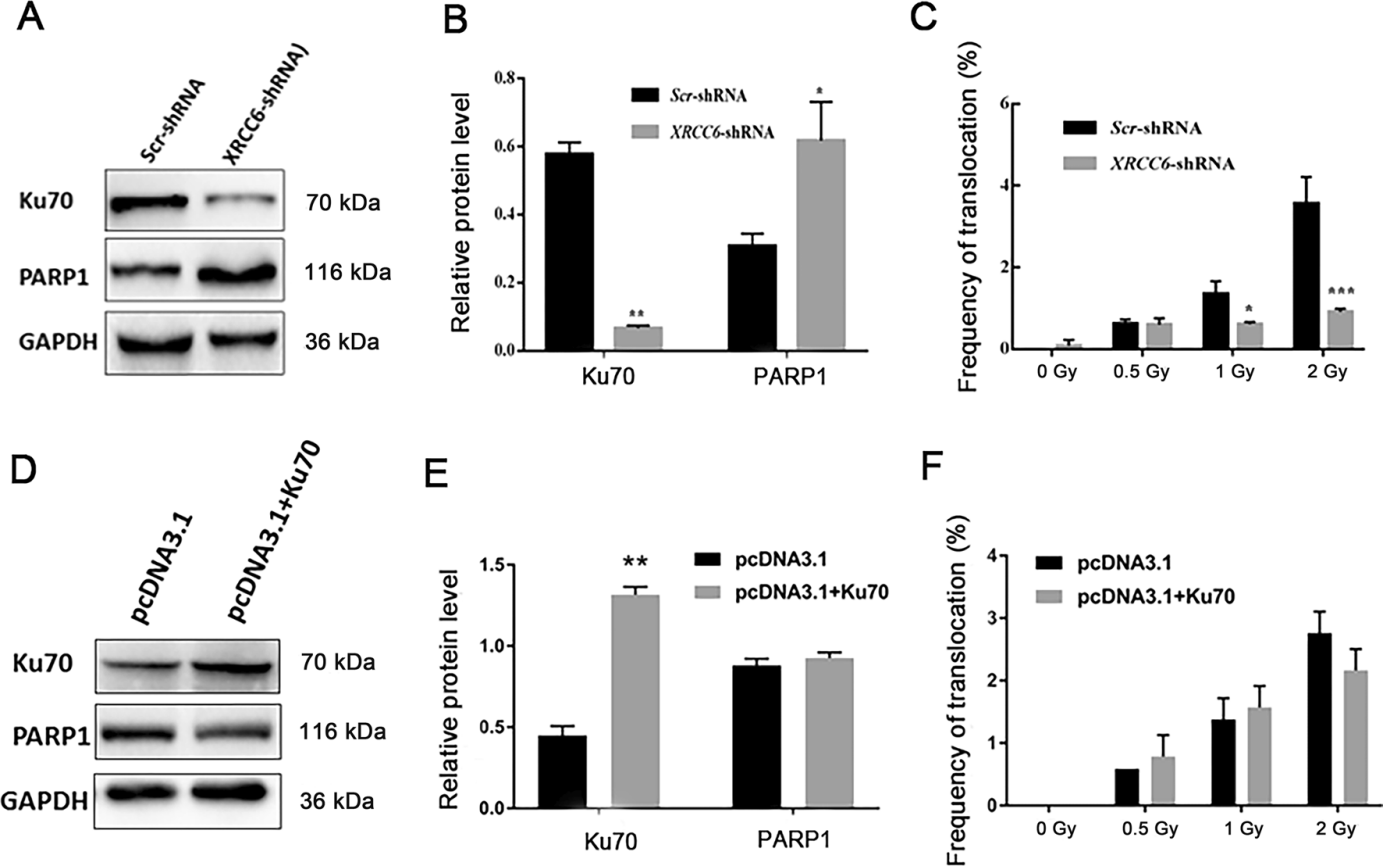
Effects of Ku70 interference and overexpression on the frequency of chromosome translocations. **A** Representative western blots images in Ku70 interference (XRCC6-shRNA) and scr-RNA (control) cells. **B** The protein expression of Ku70 and PARP1 (n = 3) in Ku70 interference (XRCC6-shRNA) and scr-RNA (control) cells. **C** Analysis of the frequency of chromosome translocations (n = 3) in Ku70 interference (XRCC6-shRNA) and scr-RNA (control) cells. **D** Representative western blots images in Ku70 overexpression (pcDNA3.1 + Ku70) and pcDNA3.1 (control) cells. **E** The protein expression of Ku70 and PARP1 (n = 3) in Ku70 overexpression (pcDNA3.1 + Ku70) and pcDNA3.1 (control) cells. **F** Analysis of the frequency of chromosome translocations (n = 3) in Ku70 overexpression (pcDNA3.1 + Ku70) and pcDNA3.1 (control) cells. *indicates that *P* < 0.05, **marks that *P* < 0.01. ***meand that *P* < 0.001. Sh, short hairpin; Scr, scrambled control

To further prove the correlation between Ku70 and chromosomal translocation, western blot was conducted to confirm the interference or overexpression efficiency and the effects on PARP1 expression. Ku70 was significantly reduced accompanied by a significant increase in PARP1 (Fig. 4a, b, *P* < 0.01 and *P* < 0.05 respectively), while PARP1 level changed rarely when Ku70 was overexpressed (Fig. 4d, e, Ku70 *P* < 0.01 and PARP1 *P* > 0.05). In order to investigate the role of Ku70 in response to chromosome translocations, the Ku70 interference or overexpression cells were treated with 0.5 Gy, 1 Gy and 2 Gy X-rays, respectively. It was observed that the frequency of chromosome translocations was significantly decreased after Ku70 interference when exposed in 1 Gy and 2 Gy X-rays (Fig. 4c, *P* < 0.05 and *P* < 0.001 respectively), and no differences were found in 0.5 Gy. We speculate that this fact could be related to the radiation dose-dependent involvement of Ku70. Zhao et al. also found Ku70 was associated with the irradiation doses [25]. The expression of Ku70 can be markedly increased after 1 Gy or 2 Gy X-rays (Fig. 1a–c, *P* < 0.05), so translocations were still remaining. However, the frequency of translocations was not significantly affected by Ku70 overexpression (Fig. 4f, *P* > 0.05).

### PARP1 inhibitor increased the expression of Ku70 and translocations after X-rays

**Fig. 5:**
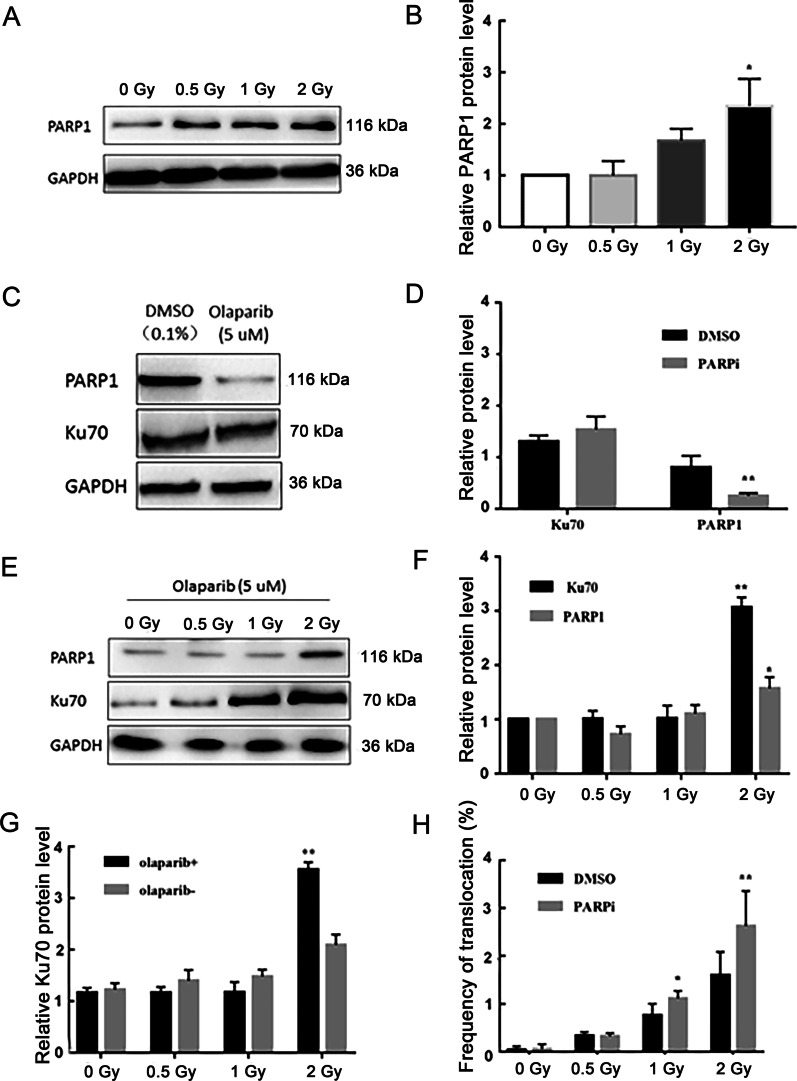
Effects of PARP1 inhibitor on the expression of Ku70 and translocations in AHH-1 cell lines. **A** Representative images of the protein expression of PARP1 in cells treated with different dose X-rays. **B** The protein expression analysis (n = 3) of PARP1 in cells treated with different dose X-rays. **C** Representative images of the protein expression of PARP1 and Ku70 in cells treated with PARPi (Olaparib). **D** The protein expression analysis (n = 3) of PARP1 and Ku70 in cells treated with PARPi (Olaparib). **E** Representative images of the protein expression of PARP1 and Ku70 in cells treated with X-rays and in combination with PARPi (Olaparib). **F** The protein expression analysis (n = 3) of PARP1 and Ku70 in cells treated with X-rays and in combination with PARPi (Olaparib). **G** The protein expression of Ku70 (n = 3) in PBLs treated with X-rays alone (Olaparib^−^) and in combination with PARPi (Olaparib^+^). **H** The frequency of chromosome translocations (n = 3) in cells treated with X-rays alone (DMSO) and in combination with PARPi (Olaparib). *stands for that *P* < 0.05, **indicates that *P* < 0.01

To further explore the effects of the PARP1 inhibitor on Ku70 and translocations, Olaparib was used. The connection of PARP1 was first observed with irradiation dose before the inhibitor was used. From Fig. 5a, b, the PARP1 expression in cells increased with the increase of X-rays dose. The expression of PARP1 decreased apparently in 3 h after being pre-treated with Olaparib (Fig. 5c, d, *P* < 0.01), and Ku70 was not significantly affected (Fig. 5c, d, *P* > 0.05). Ku70 only increased in cells treated with Olaparib after 2 Gy X-ray was accompanied by a slight increase of PARP1 (Fig. 5e, f, *P* < 0.01, *P* < 0.05). Compared with cells treated with 2 Gy X-rays alone, only Ku70 in cells treated with Olaparib in combination with 2 Gy X-rays increased significantly (Fig. 5g, *P* < 0.01). The frequency of chromosome translocations also remarkably increased in cells treated with Olaparib than cells treated with DMSO in combination with 1 Gy or 2 Gy X-rays (Fig. 5h, *P* < 0.05 and *P* < 0.01).

### Ku70 in T-ALL was significantly higher, especially in T-ALL with translocation

**Fig. 6:**
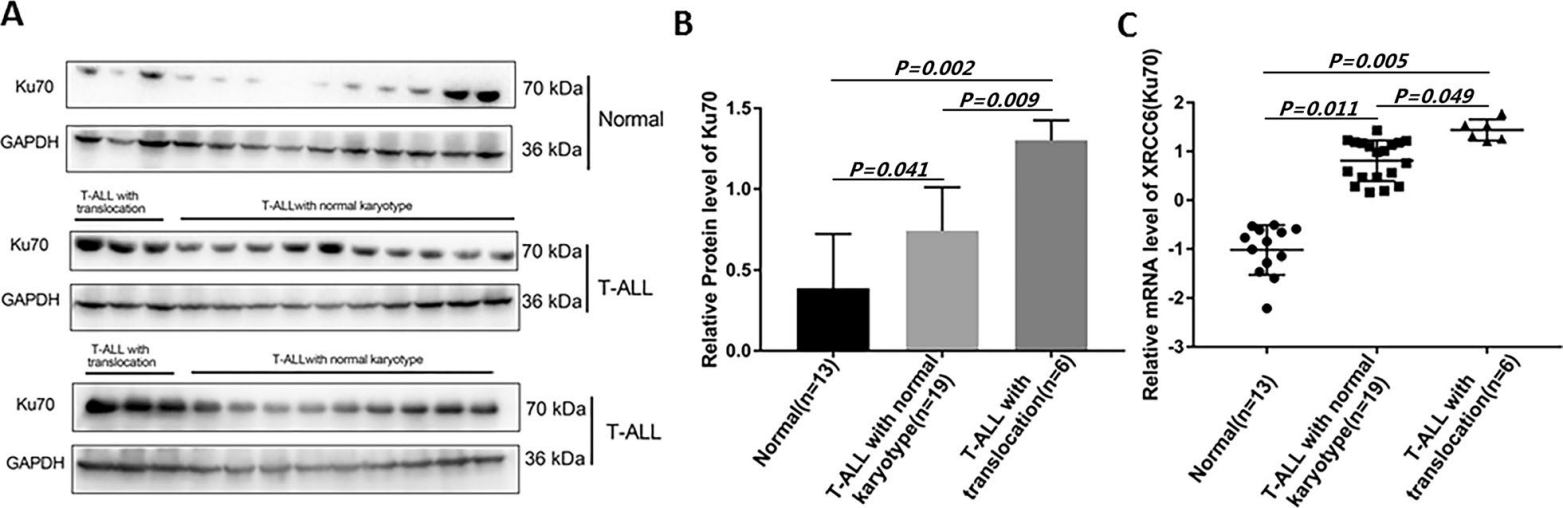
Ku70 protein level in T-ALL and the normal control. **A** Representative blots images of Ku70 in T-ALL and normal control. **B** Statistical analysis chart of Ku70 protein levels among groups. The figure illustrates that Ku70 protein levels are significantly high in both T-ALL with translocation and T-ALL with normal karyotype group compared with normal control. **C** XRCC6 mRNA levels in T-cell acute lymphoblastic leukemia samples from Xiangya hospital. X-axis represents different groups, and Y-axis is the expression of XRCC6. The scatter plots derived from RT-qPCR comparing expression of XRCC6 gene in normal control (left plots, n = 13), T-ALL with normal karyotype (middle plots, n = 19) and T-ALL with translocation (right plots, n = 6). The mRNA levels of XRCC6 are significantly higher in T-ALL than that in the control. T-ALL: T-cell acute lymphoblastic leukemia

Ku70 protein level in T-ALL (with translocation or normal karyotype) was significantly higher than that in the normal control (Fig. 6a, b, *P* = 0.002 and *P* = 0.041 respectively), and Ku70 protein level in the translocation group was significantly higher than that in normal karyotype group (Fig. 6a, b, *P* = 0.009). The same phenomenon was found in the mRNA level of Ku70 (Fig. 6c, *P* = 0.005, *P* = 0.011 and *P* = 0.049, respectively).

## Discussion

In the present study, it was first found that there was a dose- and time-response of radiation on DNA double-strand breaks (DSBs) in human peripheral blood lymphocytes (PBLs) irradiated by X-rays, and both of the expression of Ku70 and the frequency of chromosome translocations were increased with X-rays dose. A positive correlation between the expression of Ku70 and the frequency of chromosome translocation was observed in human PBLs irradiated by X-rays. The cell cycle affects DSBs rather than translocation outcome in irradiated cells. The frequency of chromosome translocations significantly decreased in Ku70-deficient cells. PARP1 slightly increased in response to DSBs irradiated by X-rays. The expression of Ku70 and the frequency of chromosome translocations increased in lymphocyte cell lines treated with PARP1 inhibitor and X-rays. Finally, our results were verified in clinical samples, and it suggested that the expression of Ku70 was significantly higher in T-cell acute lymphoblastic leukemia (T-ALL) patients than that in remission patients, especially in T-ALL with translocation. All these findings provide convincing evidence that Ku70 is closely associated with the frequency of chromosome translocation in human lymphocytes after radiation and T-ALL.

Previous studies have demonstrated that the level of Ku70 is related to the hepatocellular carcinoma prognostic factors, such as the tumor size, tumor nodule number, and distant metastasis [26]. On the other hand, clinical pathological analysis indicated that the high expression of Ku70 is associated with poor prognosis [27]. It was speculated that there was a novel mechanism of Ku70-mediated genetic alterations (such as translocation) and hematological carcinogenesis.

Translocations were probably related to the utilization of DSBs repair pathway. Sequencing translocation junctions in leukemia revealed that the translocations predominantly arose by non-homologous end-joining (NHEJ) [28]. There are 2 major NHEJ pathways: the classical pathway (c-NHEJ); and the alternative pathway (a-NHEJ) [29]. Radiation-induced translocations are usually catalyzed by PARP1 dependent a-NHEJ and defects in the c-NHEJ components increased translocation frequency [21–23], indicating that c-NHEJ normally represses translocations [30, 31]. The a-NHEJ was shown to be required for chromosomal translocations, and the PARP1 is the rate-limiting initial step of a-NHEJ [32]. However, there are still paradigms that c-NHEJ mediates translocations in human cells [33, 34]. In the present study, we also found that the c-NHEJ component Ku70 could promote translocations to some extent. We speculate that utilization of pathway may vary by cell forms. Ghezraoui et al. also reported that chromosomal translocations in a human pre-B cell line depend on c-NHEJ [34]. In addition, Ku70 might also be involved in other error-prone pathway.

Radiotherapy induces cancer cells death mainly by causing DSBs, thus resulting in chromosome instability and translocation [35]. X-ray radiation is a major therapeutic modality for cancer treatment since it kills tumor cells by inducing DSBs. In recent years, many researchers have demonstrated the key role of Ku70 in DSBs repair, while few studies focused on its role in the formation of chromosome translocation. In our study, the significant up-regulation of Ku70 indicated the active role of Ku70 in X-rays irradiated PBLs and the formation of chromosome translocation, which is consistent with Sheike et al. report that the level of Ku70 up-regulates in human peripheral blood mononuclear cells after γ-rays [36].

Neutral comet assay is mainly carried out to assess the severity of DSBs [37], and DSBs are initial events for the formation of chromosome translocations [4, 5]. Therefore, in our study neutral comet assay was used to detect the dose- and time-response of radiation on DSBs in PBLs to evaluate the formation of chromosome translocation. The result verified that it was difficult to repair more serious DSBs caused by high X-rays [38], and the frequency of chromosome translocations increased with the increase of X-rays dose within limits. To strengthen the comparison between radiation groups, the experiment was strictly controlled to ensure minimal DNA damage in the control group (0 Gy). Furthermore, considering the high sensibility for T cells to X-rays, increased DNA double-strand breaks were observed after 0.5 Gy X-ray [39]. However, the repair of minimal DSBs might be accomplished rapidly, so the DSBs could not be detected in 0.5 Gy at 72 h. These findings could also be used to evaluate the biological effects of various X-rays radiation doses on human PBLs.

Increasing studies indicated a pivotal role of chromosome translocation in the progression of tumorigenesis [40–43], and it was speculated that Ku70 might also participate in the formation of chromosome translocation. In our study, the frequency of chromosome translocations increased with the doses of X-rays, and it showed a positive correlation with the expression of Ku70, which is consistent with what Ghezraoui reported [34]. Previous researchers also explored that Ku protein regulates the end-resection of unbroken forks and HR-mediated replication restart [9], and it has an inhibitory effect on other repair pathways [44]. For example, PARP1 is suppressed by Ku70 for its high affinity to DSBs [45]. Besides, Ku protein also plays an important role in regulating DSB end-processing during HR and Alt-EJ [46]. Previous study has also demonstrated that the promotion of NHEJ results in chromosome aberrations and cell death [47]. Therefore, it was concluded that high Ku70 expression might inhibit other repair pathways and promote the error-prone repair pathway ending in translocations.

To further explore the mechanistic role of Ku70 in chromosome translocation, Ku70 expression was silenced by shRNA in AHH-1 cell lines followed by X-rays. It was observed that PARP1 slightly increased in response to DSBs, which was consistent with previous literature that X-ray irradiation produces dose-dependent increases in poly (ADP-ribose) (PAR) formation, an index of PARP1 activation [48]. Meanwhile, the frequency of chromosome translocations significantly decreased in Ku70-deficient cells. However, Ku70 overexpression did not increase the frequency of translocations. It was speculated that, with sufficient Ku70, the continued increase of Ku70 will not affect translocation.

Olaparib is one of the commonly used PAPR1 inhibitors as an antitumor drug in clinic. PARP1 inhibition could reduce the a-NHEJ frequency and the frequency of DSB-induced chromosome translocations in Ku-deficient cells [49, 50]. To further explore whether a-NHEJ is the predominant pathway involve in X-ray induced translocation or not, Olaparib was used. Our results indicated that the expression of Ku70 and the frequency of chromosome translocations increased when cells were treated with Olaparib (PARP1 inhibitor) compared with cells treated with X-rays alone, which indicated that c-NHEJ is also an important pathway involved in translocation. Previous studies have also demonstrated that Ku70/80 and PARP1 proteins compete for binding to DSBs for repair [51]. Interestingly, in Fig. 5e, PARP1 is not suppressed by Olaparib in 2 Gy X-rays, and it was found that the high expression of Ku70 or high dose X-ray seems to reduce the effects of Olaparib.

Despite sufficient powerful mastery and analysis, one of the limitations of our study might be the relatively small clinic sample size (only 38 T-ALL collected in three years), which does not allow definite conclusions. Another limitation is that only experiments in vitro were performed. More researches especially large samples combined with experiments in vivo are needed in future to focus on radiation therapy, aiming to find and manufacture inhibitors or regulate epigenetic modification of Ku70 to achieve the curative effect of tumors.

Nevertheless, this study has several strengths, including the combination of using bone marrow, peripheral blood lymphocytes (PBLs) and lymphocyte cell lines for analysis, and case–control and inclusion of patients with standard clinically defined T-ALL. The high expression of Ku70 was found in T-ALL, especially in T-ALL with chromosome translocation, and a high-frequency chromosome translocation model using PBLs was constructed by exposing in X-ray. Moreover, it was also found that the high expression of Ku70 or high dose X-ray may reduce the effects of PARP1 inhibition. These all could be conductive to guiding future researches on molecular mechanisms and therapy of T-ALL.

In conclusion, the present study suggested that Ku70 is closely associated with the frequency of chromosome translocation in human lymphocytes after radiation and T-ALL for the first time. Ku70 might be a radiation damage biomarker and a potential tumor therapy target.

## Supplementary Information

**Additional file 1: Table S1**. The details of all patients.

## Data Availability

The materials used and data sets analyzed in the current study are available from the corresponding author on reasonable requests.
