# Supplementary material for: Ku70 affects the frequency of chromosome translocation in human lymphocytes after radiation and T-cell acute lymphoblastic leukemia
Source: Radiat Oncol. 2022 Aug 19;17:144. doi: 10.1186/s13014-022-02113-3 (PMC9389784; doi:10.1186/s13014-022-02113-3)
Supplement: Supplementary file 1 — Additional file 1: Table S1. The details of all patients. [file 13014_2022_2113_MOESM1_ESM.docx]

Table S1. The details of all patients

| Sample ID | Gender | Age | Ethnicity | Occupations | Karyotype | Diagnosis |
| --- | --- | --- | --- | --- | --- | --- |
|  | Male | 10 | Han | Pupil | 46,XY, t(1;14)(p34;q11) | T-ALL |
|  | Female | 30 | Han | Worker | 46,XX,t(2;10)(q34;p12) | T-ALL |
|  | Female | 55 | Han | Worker | 46,XX, t(1;14)(p34;q11) | T-ALL |
|  | Male | 18 | Han | Student | 46,XY,t(1;4)(p34;p16) | T-ALL |
|  | Male | 36 | Han | Worker | 46,XY,t(10;14)(q24;q11) | T-ALL |
|  | Male | 43 | Han | Worker | 46,XY,t(7;10)(q35;q24) | T-ALL |
|  | Male | 15 | Han | Student | 46,XY | T-ALL |
|  | Female | 33 | Han | Worker | 46,XX | T-ALL |
|  | Female | 52 | Han | Farmer | 46,XX | T-ALL |
|  | Male | 19 | Han | Worker | 46,XY | T-ALL |
|  | Male | 29 | Han | Worker | 46,XY | T-ALL |
|  | Male | 33 | Han | Worker | 46,XY | T-ALL |
|  | Male | 16 | Han | Student | 46,XY | T-ALL |
|  | Female | 28 | Han | Worker | 46,XX | T-ALL |
|  | Female | 55 | Han | Farmer | 46,XX | T-ALL |
|  | Male | 19 | Han | Jobless | 46,XY | T-ALL |
|  | Male | 37 | Han | Worker | 46,XY | T-ALL |
|  | Male | 44 | Han | Worker | 46,XY | T-ALL |
|  | Male | 14 | Han | Student | 46,XY | T-ALL |
|  | Female | 30 | Han | Worker | 46,XX | T-ALL |
|  | Female | 15 | Han | Student | 46,XX | T-ALL |
|  | Male | 40 | Han | Worker | 46,XY | T-ALL |
|  | Male | 38 | Han | Jobless | 46,XY | T-ALL |
|  | Male | 55 | Han | Worker | 46,XY | T-ALL |
|  | Male | 48 | Han | Worker | 46,XY | T-ALL |
|  | Female | 36 | Han | Farmer | 46,XX | Remission |
|  | Male | 43 | Han | Worker | 46,XY | Remission |
|  | Female | 19 | Han | Student | 46,XX | Remission |
|  | Female | 30 | Han | Worker | 46,XX | Remission |
|  | Male | 55 | Han | Worker | 46,XY | Remission |
|  | Male | 28 | Han | Worker | 46,XY | Remission |
|  | Male | 37 | Han | Worker | 46,XY | Remission |
|  | Male | 11 | Han | Pupil | 46,XY | Remission |
|  | Female | 60 | Han | Retired | 46,XX | Remission |
|  | Female | 25 | Han | Student | 46,XX | Remission |
|  | Male | 48 | Han | Worker | 46,XY | Remission |
|  | Male | 36 | Han | Worker | 46,XY | Remission |
|  | Male | 43 | Han | Worker | 46,XY | Remission |

T-ALL: T cell acute lymphoblastic leukemia.
